# Effects of the Herbal Product Curcumin on Cardiomyocytes in Micromass Culture and the Potential Role It May Play in Pregnancy and Development

**DOI:** 10.7759/cureus.51132

**Published:** 2023-12-26

**Authors:** Priyan Magan, Margaret Pratten

**Affiliations:** 1 School of Life Sciences, University of Nottingham, Nottingham, GBR

**Keywords:** adverse pregnancy outcome, herbal compounds, herbal medications, embryo growth, chick embryo

## Abstract

Introduction

Herbal medicine (HM) consumption during pregnancy has been on the rise in many parts of the world. Curcumin is a proven antioxidant and anti-inflammatory herb component, having demonstrated efficacy in alleviating various diseases. However, there is conflicting evidence with regards to its effect on pregnancy. We assess the safety profile of the main component of turmeric, curcumin, during pregnancy. Furthermore, to investigate curcumin in combination with known teratogen ethanol to identify any protective effect that curcumin might exert.

Method

Embryonic chick cardiomyocytes in micromass culture were treated with varying concentrations of curcumin. Three endpoints were used to determine the effect of curcumin on these cells: contractile activity (morphological score), cell viability (resazurin assay), and total protein content (kenacid blue assay).

Results

Curcumin demonstrated cytotoxicity at the highest tested concentrations (10-20μM) by significantly reducing cell activity and total protein. The results of morphological scoring suggest that repeated investigations would have revealed the teratogenic potential of curcumin. Lower concentrations (50nM) of curcumin were comparable to the control. The combination of a non-toxic concentration of curcumin with ethanol revealed additive toxicity.

Conclusion

It seems unlikely that curcumin will adversely affect the embryo at low doses due to issues of bioavailability. The findings of cytotoxicity and possible teratogenicity at high concentrations are a concern. Due to the limited information available regarding curcumin metabolism in human embryos, advancements in curcumin delivery systems, and the high likelihood of overconsumption, further in vivo research using animal models is required.

## Introduction

Curcumin, the principal component of turmeric, has been used as a medicinal and culinary plant for years. Numerous studies have demonstrated the potential of curcumin against certain pathological states via its anti-inflammatory, antioxidant, and anticarcinogenic properties. Through the inactivation of NF-kB, curcumin can downregulate cytokines, thus reducing inflammation, as demonstrated in several mouse models [1]. There is evidence to suggest a possible benefit, specifically in pregnancy-related conditions such as gestational diabetes and pre-eclampsia, as well as protection afforded against cytotoxic and teratogenic agents [2,3].

This study aimed to identify any beneficial or toxic effects that curcumin may exert during pregnancy, i.e., any developmental or reproductive toxicity. The effect of curcumin on chick embryonic cardiomyocytes grown in micromass culture was studied. The basis of in vitro micromass (MM) culture is that teratogens will inhibit there-differentiation' of primary embryonic cells. The vital early processes seen in embryonic cells, such as cell differentiation, proliferation, and communication, stay uniform across different species. Hence, observations in chick/rat embryonic cells may correlate to similar outcomes in human embryos [4]. This study also investigated the effect of curcumin on chick cardiomyocytes when administered alongside a known teratogen, ethanol. This was to assess if there was a protective effect offered by curcumin, as suggested in mouse models, or an additive toxicity [5].

Utilizing chick embryos removed the need to kill the mother, and since the embryo had not reached the midpoint of its development, no Home Office license was required. Pratten et al. noted that the results at the end point might be similar to those seen in vivo, as chick embryos may naturally grow without the presence of the mother [6].

The micromass (MM) system has been used to assess embryonic cells of midbrain, limb or cardiac origin, as all undergo significant embryonic development within days - reducing tissue waste [4]. The chick heart demonstrates high teratogenic sensitivity during days 2-6 and considerable growth relative to the whole embryo [7]. This made cardiomyocytes an appropriate cell type with which to treat with curcumin. Various other papers have demonstrated the cardio-protective role of curcumin [8,9]. To the best of our knowledge, there was no literature indicating curcumin-induced toxicity to cardiomyocytes.

Previous studies in this laboratory have demonstrated the presence of cardiac muscle cells as opposed to skeletal muscle cells in micromass culture [4]. In addition, more detailed immunocytochemical studies in similar cultures performed by Ahir et al. demonstrated the presence of predominantly cardiomyocytes and the role of connexins in the mediation of the effects of caffeine on chick cardiomyocytes in micromass culture [10]. Immunohistochemistry was used to show that cells within the chick MM culture employed in this study primarily yielded cardiomyocytes, showing positive staining for cardiac-specific α- and β-myosin heavy chains and cardiac troponin T antibodies. The expression of connexin (Cx40, Cx43, and Cx45) is a recognized factor in the development of the cardiac system. Cx43 was the first gap junction protein found in heart tissue, and it is expressed in virtually all myocytes of the working myocardium [11].

## Materials and methods

Cell optimization, seeding, flooding

For a full list of solutions, chemicals, drugs, equipment, and consumables, refer to Appendix 1. White Leghorn chicken eggs (Henry Stewart & Co. Ltd., UK) were initially kept in a cold incubator at 12°C to halt development for a maximum of two weeks. When required, eggs were placed in a warm incubator (37°C, 90% humidity, 5% CO2) for a total duration of five days. Eggs were placed on an automatic rotator, which would help maintain them and provide optimal conditions. The day of initial incubation was noted as day ‘0’. 

On day five (explantation day), the surface was cleaned with Trigene. Forceps, the hood, and the eggs (removed from the incubator and placed in the hood) were all sprayed with Industrial Methylated Spirits (70% IMS). It was crucial to wipe away all IMS after spraying to prevent the risk of embryonic exposure during explantation.

The curved, blunt end of the forceps was used to crack the eggshell initially, following which the blood vessels and membrane were removed. The embryo was then carefully removed from the egg using the curved end of the forceps. Care was taken not to touch the beating heart with the forceps at any point, and instead, the embryo was held by the limb. A microscope was used to aid in the identification of the heart, which could be seen as red and beating. The heart could be distinguished from other blood vessels due to its classic proximity to the eye. Embryonic hearts were dissected out using bent and pointed forceps in a petri dish containing enough HBSS to fill the base of the dish.

The hearts were collected in a bijou tube, which contained a 50:50 composition of horse serum (3ml) and HBSS (3ml). Throughout heart dissection, the bijou tube would be placed in a beaker of ice in order to reduce cardiomyocyte death. Hearts were then washed with 5ml of HBSS on three separate occasions. Following the last wash, HBSS was removed, and 2ml of 2.5% trypsin/EDTA was added. Hearts were trypsinized in a CO2 incubator at 37°C for 20 minutes. Every 5 minutes, the hearts were agitated for 30 seconds to aid in the formation of a homogenous solution of cardiomyocytes. Upon completion of the 20 minutes, dissociation of the cells was aided via delicate pipetting up and down. This suspension was then carefully transferred to a 15-ml centrifuge tube. A cell culture medium was prepared fresh at the start of each week using DMEM (450 mL), 50 mL FBS, 5 mL L-glutamine (200 mM), and 5 mL 50μg/mL penicillin/streptomycin. This medium was added to the cell suspension to quickly neutralize trypsin activity. Centrifugation of this suspension followed for 5 minutes at 1500 rpm. The supernatant was aspirated, and the pellet formed at the bottom of the tube was then resuspended in 500 μl of medium. A cell count was then performed, combining 5μl of Trypan blue and 5μl of the cell suspension onto a counting slide. The cell density was always adjusted to 3 x 106 cells/mL. Cell density of 3x106 cells/ml was used, as this was identified as optimal in previous studies [10]. Cells were seeded as 20μl drops into a 24-well plate (Nunclon, UK). Cells were given 2.5 hours to attach to the base of each well in the C02 incubator. The wells were then flooded with 500 μl of the warmed DMEM-based medium prepared earlier. This point in time was regarded as '0 hrs'. The flooded plates were then left for 24 hours in incubation again (37°C).

Curcumin-only and combination treatment 

**Figure 1:**
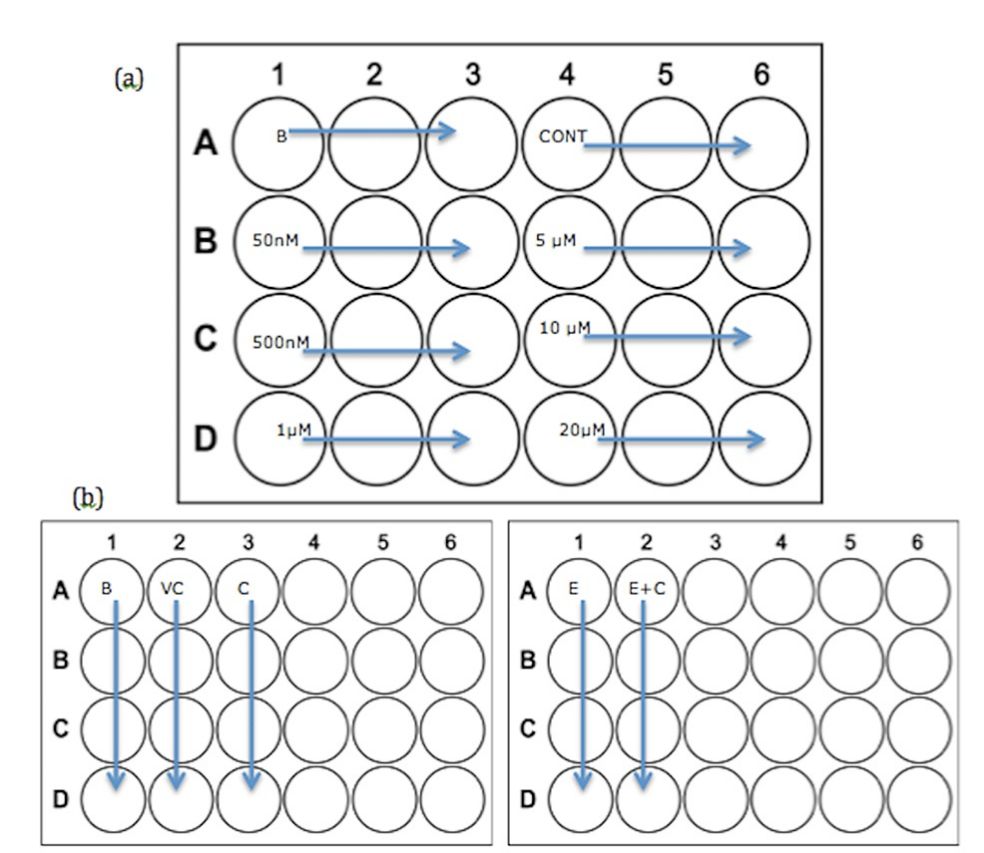
Layout of a 24-well plate for each of the experiments a) Curcumin treatment experiments. All concentrations are in either μM (micromol) or nM (nanomol). B: Blank, CONT: Control (treated with vehicle control) b) Curcumin/Ethanol Experiments (required 2 plates per experiment) B: Blank, VC: Vehicle control, C: Curcumin (5μM), E: Ethanol (75μl/ml), E+C: Combined ethanol (75μl/ml) with curcumin (5μM)

Upon completion of the 24 hours (after morphological scoring, as described below), a further 500μl comprised of medium and curcumin was added. A curcumin stock solution was formed using 5 mg of curcumin in 120μl DMSO, which, in turn, was diluted to enable testing of the following curcumin concentrations: 50nM, 500nM, 1μM, 5μM, 10μM and 20mM.3 control wells (seeded) and 3 blank wells (not seeded) were included in each plate. Ultimately, all wells were flooded on two occasions (2.5 hours after seeding and 24 hours after initial flooding). Test chemical wells were treated with their respective concentrations of curcumin, while blanks and controls were always flooded using the DMSO-based vehicle control (Figure 1a). Combination experiments were conducted over two plates, as ethanol would otherwise evaporate and damage cell culture in control or cucumber-only wells (Figure 1b). 5μM curcumin and 75μl/ml ethanol were prepared in the same way as indicated above. This concentration of curcumin was chosen because it was the highest non-toxic concentration. Vehicle control was prepared as described above. 

Measurements and assays

Cell Morphology

A morphological scoring system was used, as indicated in Table 1, the same system previously used by others [12]. This scoring system was based on the contractility/’beating’ of the cells when observed under a light microscope. Cells were inspected for their contractile activity 24 hours after initial 500μl flooding (before curcumin treatment) and then twice after curcumin treatment (at 48 hours and 144 hours).

**Table 1:** Morphological scoring system Morphological scores numbered 0-3 with their corresponding contractile activity

Numerical morphological score	Contractile activity
0	No contractile activity
1	One or two contracting foci
2	Numerous contracting foci
3	Entire plate contracting

Resazurin Assay

The resazurin assay was conducted to assess cell viability/cell activity. The principle behind this assay is that the oxidized ‘resazurin’ (blue) undergoes reduction, forming a noticeably pink and fluorescent ‘resorufin’. This irreversible reduction reaction occurs when living cells are present to take up the resazurin. Essentially, greater cell activity should be associated with greater resorufin production, and this can be measured based on fluorescence [13].

This assay was conducted immediately after final scoring (144 hours after explanation). The culture medium was aspirated very carefully from all wells in the plate, ensuring that the cells were not removed in the process. To replace the aspirated medium, 500μl of pre-warmed 10μg/ml resazurin in HBSS was added to every well. The plate was then left in the incubator for 1 hour at 37°C, 5% CO2. The optical density was then measured using a FLUORstar Galaxy fluorescence plate reader. The wavelength settings were: an excitation filter of 530 ± 10nm, an emission filter of 590 ± 12.6, and a gain of 60.

Kenacid Blue Assay

The Kenacid blue assay, which takes place immediately after completion of the resazurin assay, is a way of measuring total protein content in each well. The protocol, initially described by Knox et al. (1986), is based on the idea that cell loss is associated with protein content reductions [14].

All wells were initially aspirated, and instead, 300μl kenacid blue fixative (500ml ethanol, 490ml distilled water, and 10ml acetic acid) was added. The plate was then left overnight in a refrigerator at 4°C. The next day, a fresh preparation of kenacid blue working solution was made by adding 6 ml of glacial acetic acid to 44 ml of kenacid blue stock solution (400 ml of kenacid blue dye powder, 250 ml of ethanol, and 630 ml of distilled water). The existing fixative solution was carefully aspirated, and 500 μl of newly made kenacid blue working solution was added to each well. The plates were then placed on the shaker at speed 3 for two hours. On completion, the working solution was aspirated, followed by the addition of 400 μl of kenacid washing solution to each well (100 ml ethanol, 50 ml acetic acid, and 850 ml distilled water). This washing solution was then itself aspirated and replaced with another 400 μl washing solution to remove any excess staining. To ensure thorough rinsing, the plate was placed on the shaker for 20 minutes at speed 3 at this point. The washing solution was then replaced with 400 μl of desorbing solution (700 ml ethanol, 98.15g potassium acetate, and 300 ml distilled water). Agitation for one hour at speed 6 on the shaker followed. 100μl from each well was transferred to a 96-well plate, and optical density was measured using the Multiskan Ascent plate reader (‘Ascent’ software). Settings: reference filter: 405 nm, reading filter: 570nm. 

Statistical analysis

The data from all endpoints was organized into Microsoft Excel, and analysis was conducted via Graphpad Prism 6. The analysis was conducted as such (p<0.05 for significance in both): A) Resazurin and kenacid blue (parametric): one-way ANOVA (post-hoc: Dunnett’s test). B) Morphological scoring (non-parametric) Kruskall-Wallis (post-hoc: Dunn’s test).

## Results

Test chemical: curcumin

Morphological Scoring

**Figure 2:**
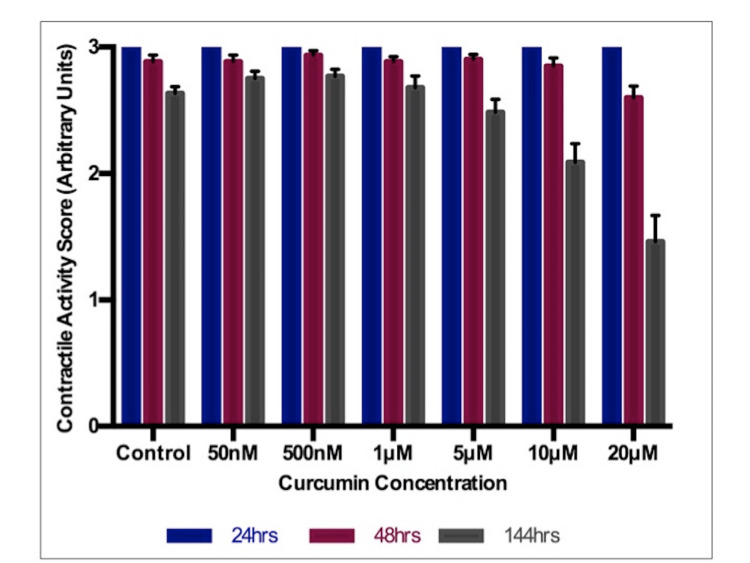
Morphological score Contractile activity (arbitrary units), Curcumin concentration in μM (micromol) or nM (nanomol)

At 24 hours, there is no difference in contractile activity observed at any concentration, as expected before curcumin administration. For 48-hour scoring, there is only a slight reduction in contractile activity at the highest concentration (20 μM), albeit not statistically significant (Figure 2). Typically, at 48 hours of scoring, it is seen that various beating foci are starting to coalesce, forming the beginnings of a sheet. At 144 hours, contractile activity stays relatively unchanged for 50 nM and 500 nM curcumin concentrations, compared to the control. In fact, the mean score shows a slight increasing trend from control (mean: 2.633) to 50 nM (mean: 2.750) to 500 nM (mean: 2.768), although neither of these is significant. As concentrations increase above 500 nM, there is a decreasing pattern in morphological scoring. 

Cellular Activity

**Figure 3:**
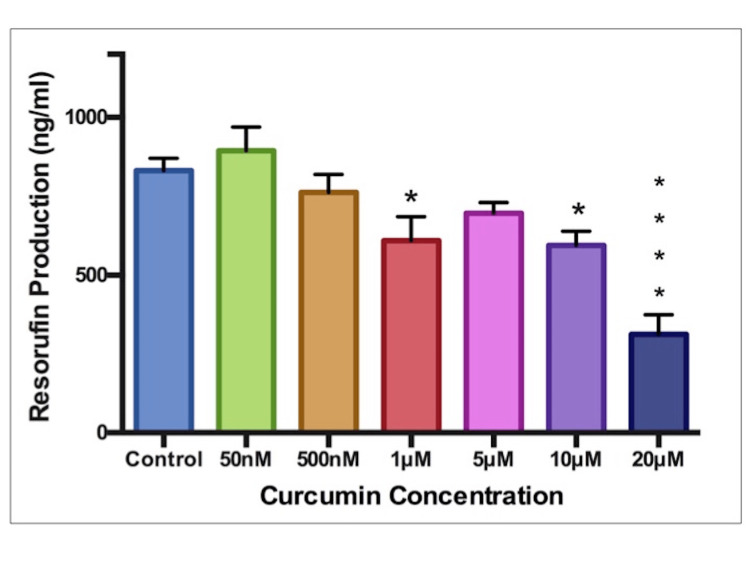
Cell activity Measured as Resorufin production (ng/ml). Curcumin concentration in μM (micromol) or nM (nanomol). * indicates statistical significance.

Cellular activity showed a decreasing tendency as curcumin concentration was increased (Figure 3). The largest, most significant decrease occurred at the highest tested concentration of 20μM (p<0.0001). Some significant reduction was also found at 1μM and 10μM concentrations (p<0.05).

Total Protein

**Figure 4:**
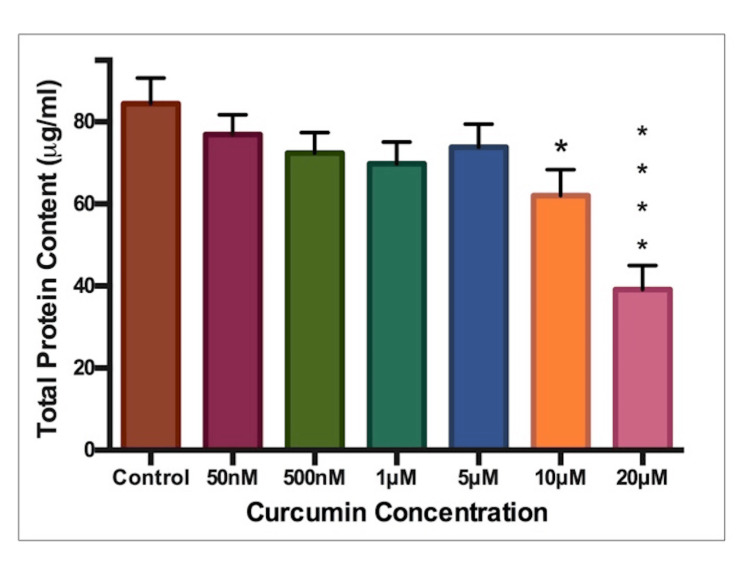
Total protein content Total protein content (μg/ml). Curcumin concentration in μM (micromol) or nM (nanomol). * indicates statistical significance.

Total protein content also showed a declining trend (Figure 4). There were significant reductions in protein at the highest concentrations of 20 μM (p<0.0001) and 10 μM (p<0.05).

Curcumin and ethanol combination

Determining Concentrations

Reference back to curcumin results indicated that the highest, non-toxic concentration was 5 μM, as it showed no significance in any endpoint. The concentration of ethanol used was 75 μl/ml, as this was in line with past studies [12]. At this concentration, there is definite toxicity in the cardiomyocyte culture, but not all the cells are killed, enabling a degree of recovery by 144 hours.

Morphological Scoring

**Figure 5:**
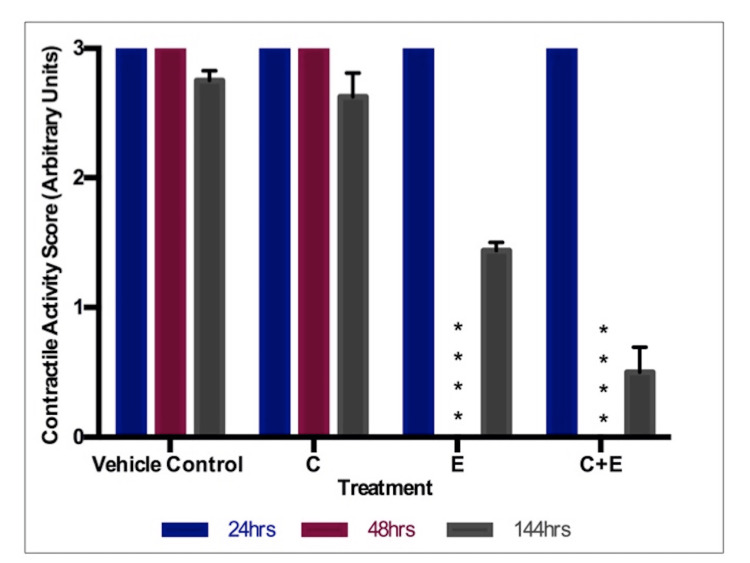
Morphological score: 5μM curcumin and 75μl/ml ethanol Contractile activity (arbitrary units). C: Curcumin (5μM), E: Ethanol (75μl/ml), E+C: Combined ethanol (75μl/ml) with curcumin (5μM). * indicates statistical significance.

For 48 hours, there was a significant reduction between the ethanol-only treatment and the ethanol/curcumin-combined treatment. In both cases, contractility was completely inhibited. 144-hour scoring showed that ethanol alone and ethanol/curcumin combined treatments reduced contractility compared to controls, yet neither was significant. The graph (Figure 5) does appear to show, however that combined treatment had a greater ability to reduce contractility at 144 hours.

Cellular Activity

**Figure 6:**
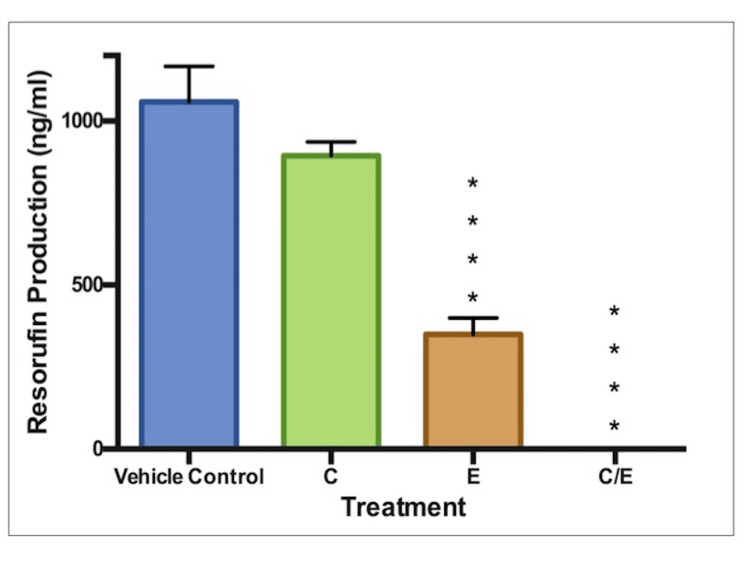
Cell activity: 5μM curcumin and 75μl/ml ethanol Measured as resorufin production (ng/ml). C: Curcumin (5μM), E: Ethanol (75μl/ml), E+C: Combined ethanol (75μl/ml) with curcumin (5μM). * indicates statistical significance.

In Figure 6, we see that ethanol and ethanol/curcumin treatment both significantly reduced cell activity (p<0.0001). However, the magnitude of the ethanol/curcumin combination was greater, as it indicated complete inhibition of cell activity.

Total Protein

**Figure 7:**
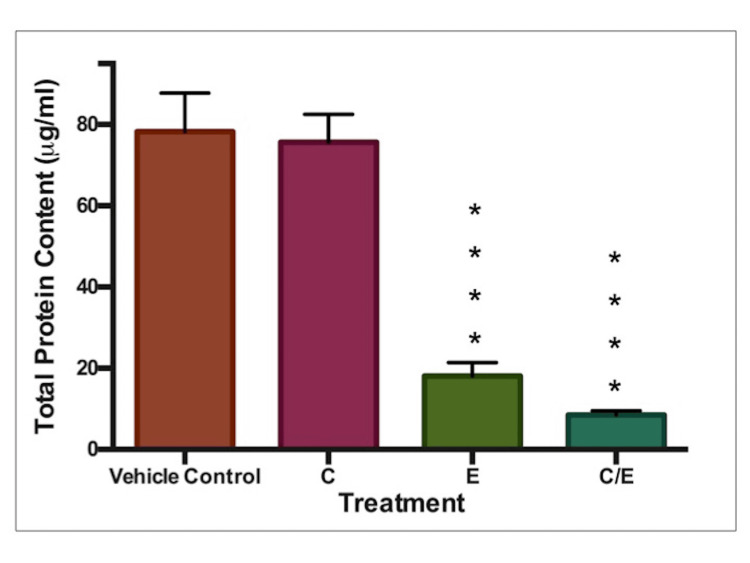
Total protein content: 5μM curcumin and 75μl/ml ethanol Total protein content (μg/ml). C: Curcumin (5μM), E: Ethanol (75μl/ml), E+C: Combined ethanol (75μl/ml) with curcumin (5μM). * indicates statistical significance.

Similar to the resazurin assay, both ethanol and ethanol/curcumin combination treatments caused significant decreases in total protein (p<0.0001), as seen in Figure 7.

## Discussion

Morphological scoring

The first endpoint, morphological scoring, showed no significance at any concentration; thus, it cannot be said for certain that curcumin is teratogenic. The cardiomyocytes, which under normal conditions will spontaneously beat, were each isolated so that they no longer beat. When placing a cluster of these non-beating cardiomyocytes in close proximity, without treatment, these cells 're-differentiate', coming together and regaining contractility. Scoring essentially measures this process, upon which curcumin had no statistically significant impact. It was unusual to not attain significance for this endpoint for various reasons. First, the dose-dependent downward trend in contractile activity observed at 144-hour scoring is clear (Figure 2). There is a definite reduction in contractile activity at 20 μM (144 hrs). It may be that through further experimental repeats to increase the sample size, the highest concentrations, at least 10-20 μM, may show statistical significance in morphological scoring. Further repeats are, therefore, likely to indicate the teratogenic potential of curcumin.

Cytotoxicity

The resazurin and kenacid blue assays both indicated that the two highest tested concentrations (10μM and 20μM) significantly reduced cell activity and total protein, respectively. Therefore, it would follow that curcumin, at high doses, exerts cytotoxic effects. A possible dose-dependent toxicity can be seen via the downward-sloping trends in cell activity and protein (Figures 3, 4). This dose-dependent observation coincides with findings in zebrafish embryos whereby reduced survival and hatching rates were observed at concentrations above 7.5 μM [15,16]. Toxic, dose-dependent relationships have also been indicated [17,18]. This was unusual, as no significance was observed in any other endpoint at this concentration. As this does not fit the trend seen in the results, this finding was deemed an anomalous result.

In comparison to existing literature, Chen et al. found oocyte maturation to decrease at 20 μM, while concentrations of 24 μM were required to reduce cell number in mouse blastocysts [19,20]. Both of these findings agree with the toxicity observed at 20 μM. Huang et al. noticed a reduction in blastocyst development at concentrations of just 6 μM [17], while zebrafish studies found 7.5 μM to be the level at which embryonic survival was significantly reduced [15]. Both of these results are at a similar level to the other toxic concentration reported here, 10 μM. The toxic concentrations identified in this study are in line with studies previously conducted at the embryonic or pre-embryonic stage. These are the best available comparators, as no previous study has focused extensively on the effects of curcumin on embryonic cardiomyocytes.

Apoptotic mechanisms may be responsible for some of the findings of embryotoxicity, as shown when curcumin was found to stimulate apoptosis in the chondrogenic competent cells of chick limb buds [21]. A definite decrease in Akt activation was observed, a protein known to promote cell survival. Curcumin treatment also drastically increased actin stress fibers. Given that this study involved myocytes and, therefore, actin fibers, the mechanisms of toxicity indicated by Kim et al. could be relevant to this investigation [21]. Chen et al. concluded that the injurious effects of curcumin in mouse blastocysts were mediated via mitochondria-dependent apoptotic mechanisms and the generation of reactive oxygen species, and they also indicated pro-apoptotic influences of curcumin [19]. Treatment at the oocyte stage prevented maturation at that stage while also initiating inappropriate apoptosis at the blastocyst stage. An alternative explanation for embryotoxicity could be in the form of meiotic division and mitotic cleavage disruptions, which are believed to be due to abnormal spindles. Blakemore et al. deduced that curcumin treatment increased mitotic spindle irregularity and, as a result, caused mitotic inhibition in a variety of cell types [22]. The same study also found that curcumin provoked G2/M arrest in every cell lineage tested. Gong et al. also found curcumin to arrest the cell cycle at that same point in human fetal retinal pigment epithelial cells, confirming this as a possible cause of embryotoxicity [23]. Bielak-Zmijewska et al. discovered that curcumin provoked the stalling of meiotic resumption [24]. The final mechanism for embryotoxicity is provided as reasoning for the mortality observed in curcumin-treated zebrafish [15,25]. The importance of controlling calcium is well known, and the sarco/endoplasmic reticulum Ca2+ ATPase (SERCA) is fundamental to maintaining cellular calcium concentrations. These pumps have been shown to undergo inhibition in the presence of curcumin [26]. It has been noticed that the effective dose concentrations required for this pump inhibition were similar to the doses of curcumin that reduced zebrafish survival [15]. In addition, ‘SERCA2a’ is the isoform of the pump found in cardiac cells [26]. Wu et al. observed reductions in heartbeat in curcumin-administered larvae, which might possibly link to a curcumin-driven SERCA2a inhibition [15]. This explanation of embryotoxicity has some validity, particularly as there is evidence of the role played by SERCA2 in zebrafish [27]. It would be of significant interest in future studies to explore whether such mechanisms play a role in the effects of curcumin on chick cardiomyocytes in micromass culture.

Curcumin benefits 

Even though the potential for curcumin to be toxic at higher levels has been discussed, it still may be beneficial at low concentrations. For instance, this study reported that concentrations of 50 nM and 500 nM were comparable to controls, demonstrating no significant difference at any end point. In fact, both of these concentrations appear superior to the control at 144-hour scoring (Figure 2), while 50 nM also appears above the control in the cell activity assay (Figure 3). Although these differences are marginal and not statistically significant, this could indicate some beneficial role of curcumin in improving re-differentiation and cell activity in embryonic cells at low concentrations. This hypothesis is further supported by Kim et al., in which a low concentration (500 nM) of curcumin was found to stimulate the greatest neural progenitor cell proliferation in mouse embryos [28]. Further investigations would be required in order to validate this. Additionally, various existing literature concludes that whether curcumin yields positive or negative effects depends upon the presence of a stressor, which it can then combat (such as high glucose, blood pressure, teratogen, etc.) [29].

An ethanol/curcumin combination was one of interest, particularly since Zhang et al. had previously described the protection afforded by curcumin in pre-natal alcohol exposure [30]. Figures 5-7 show that both ethanol treatment and ethanol/curcumin combination treatment caused significant reductions in all three endpoints. The combined treatment produced more pronounced effects. Curcumin does not appear to have provided any protective effects against the known teratogen ethanol. 5 μM curcumin was the highest non-toxic concentration in its own right. However, in combination with ethanol, some interaction or additive toxicity occurs. This finding suggests that a curcumin/ethanol combination could pose a greater risk to the embryo than alcohol consumption alone. Further experiments using a range of ethanol and curcumin concentrations are required to investigate this.

Further research

Repeating this investigation, with a particular focus on lower concentrations and the curcumin/ethanol combination, has been discussed. These investigations could involve some form of flow cytometry or Western blot analyses to identify proteins involved in cell survival, thus enabling identification of the mechanisms involved in curcumin action. Regarding the limitations of this study, curcumin administration in vitro does not reflect physiological conditions. In addition, chick embryos, as tested in this study, are likely to have variability in their metabolism of curcumin compared to human embryos. Another factor to consider is bioavailability, which is variable across different formulations of curcumin available to consumers. Variability and uncertainty in bioavailability may limit the ability for concentrations described in this study as toxic to be reached in the embryonic system. As there is conflicting evidence regarding curcumin-induced embryotoxicity, further in vivo research in animal models with ethical and considered methodology is required. Only then may a 'safe dose’ be predicted for human embryos.

## Conclusions

Consumption of the key component of turmeric, curcumin, does not raise alarm at low doses in pregnant women. However, the principal issue is that as curcumin delivery systems are improved, the cytotoxic and teratogenic capabilities of this herbal medicine, as demonstrated in this study, will pose a greater risk to embryonic development. Further to this, pregnant women risk exceeding the recommended doses of this ‘natural’ product, whether for pregnancy-related issues such as hypertension or simply to improve embryonic health through antioxidant and anti-inflammatory action. Consumption in excess of the recommended dose could expose the limited metabolism in human embryos. The evidence surrounding the role of curcumin in embryonic development is very conflicting, while the multiplicity of its downstream targets could result in unanticipated effects in the embryo. Hence, it can be concluded that high-dose curcumin and curcumin/ethanol combinations may be risk factors in embryonic development.

## Appendices

Appendix 1

Chemicals and Solutions

Cayman Chemical (Ann Arbour, USA): Curcumin

Sigma-Aldrich (Poole, Dorset, UK): Penicillin/streptomycin, Dulbecco’s Modified Eagles culture medium (DMEM), L-Glutamine, Ethanol, Trypan blue, Dimethylsulphoxide (DMSO), Resazurin, Kenacid blue powder, horse serum, 2.5% Trypsin/EDTA, Hank’s Balanced Salt Solution (HBSS), Fetal Bovine Serum (FBS), Acetic acid

Medichem International (UK): Trigene surface disinfectant

BDH (UK): Glacial acetic acid

Fisher scientific (UK): Potassium acetate

Other Equipment and Software

Cold incubator and C02 incubator - Sanyo (Japan)

Warm incubator - ChickTec (UK)

Working hood (Class 1 sterile laminar flow) - Faster (Italy)

Working hood (Class 2 laminar flow) - Walker (UK)

Bijou tubes and 50ml universal tubes- Sterilin (UK)

15ml centrifuge tube - Falcon (UK)

Pipettes - (Finnpippette, UK)

Pipette tips - Sarstedt (UK)

Centrifuge - Fisons and Sigma (UK)

Cell counting machine - Hawksley (UK)

24-well plate and 96-well plate - Nunclon (UK)

Light Microscope for scoring - Nikon (Japan)

Microscope for Explantation - Leica (UK)

FLUORstar Galaxy fluorescence plate reader - BMG Lab Technologies, Buckinghamshire (UK)

Multiskan Ascent Plate Reader - Thermo scientific (UK)

Forceps (curved, bent and pointed) and 90mm petri dish - A. Durmount (Switzerland)

C02 - British Oxygen Company

Prism 6 statistical software  - Graphpad  (USA)
